# New species of caddisflies (Trichoptera, Ecnomidae, Polycentropodidae, Psychomyiidae) from Mekong tributaries, Laos

**DOI:** 10.3897/zookeys.962.52759

**Published:** 2020-08-20

**Authors:** Pongsak Laudee, Kriengkrai Seetapan, Chanda Vongsombath, Hans Malicky

**Affiliations:** 1 Department of Fishery and Costal Resources, Faculty of Science and Industrial Technology, Prince of Songkla University, Surat Thani Campus, Muang District, Surat Thani Province, Thailand Prince of Songkla University Surat Thani Thailand; 2 School of Agriculture and Natural Resources, University of Phayao, Tumbol Maeka, Muang District, Phayao Province, Thailand University of Phayao Phayao Thailand; 3 Faculty of Environmental Sciences, National University of Laos, Dong Dok Campus, Vientiane, Laos National University of Laos Vientiane Laos; 4 Sonnengasse 13, A-3293 Lunz am See, Austria Unafiliated Scheibbs Austria

**Keywords:** aquatic insects, biodiversity, Oriental Region, taxonomy

## Abstract

Four new species of caddisflies are added to the fauna of Mekong River, Laos. Described and illustrated are *Ecnomus petchanaae***sp. nov.** and *E. boonsawaengae***sp. nov.** (Ecnomidae), *Polyplectropus proukaewi***sp. nov.** (Polycentropodidae), and *Psychomyia srichanai***sp. nov.** (Psychomyiidae) from tributaries of the Mekong River, Laos. *Ecnomus petchanaae***sp. nov.** can be distinguished by the characters of the superior appendages, which are slender and club-shaped in lateral view. In *E. boonsawaengae***sp. nov.**, the inferior appendages are tubular with a concave incision subapically and truncated apex. *Polyplectropus proukaewi***sp. nov.** has the distal process of the dorsal branch of the inferior appendage close to the base of the inferior appendages and the length of the process is half of inferior appendages. *Psychomyia srichanai***sp. nov.** can be distinguished by the structure of the dorsal branches of the harpagones and apical sclerite. The outer branches of the harpago in the latter species are bifurcated and bent 90° degrees outward, and the inner dorsal branches of harpago are curved outward. The apical sclerite is indiscernible.

## Introduction

The Mekong River, with a length of 5,400 km is the 12^th^ longest river in the world and passes through six countries, originating from China, through Myanmar, Laos, Thailand, Laos, Cambodia, and finally Vietnam (Mekong River Commission 2010a, 2010b). Its river basin is among the most diverse riverine systems of the world, where 367 new species were recently found, including 24 new species of fish and 21 new species of amphibians (World Wild Fund 2014). The Mekong River is in the Oriental Region where diversity of Trichoptera is generally high (de Moor and Ivanov 2008), however, so far this aquatic insect order has not been intensively studied along its course.

Trichoptera (or caddiflies), among the holometabolous aquatic insects, are one of the largest groups of aquatic insects contributing to many aspects in an aquatic ecosystem as secondary consumers, tertiary consumers, or predators (Dudgeon 1999). Adult Trichoptera occupy terrestrial or riparian zones along aquatic habitats. The larval stages are exclusively aquatic (Holzenthal et al. 2007). More than 5,854 species of Trichoptera have been described in the Oriental Region (Morse et al. 2019). Moreover, publications in the last 10 years on Trichoptera in the Oriental Region have recorded more than 3,000 species (Morse 2016). There are several important contributions to the knowledge on caddisfly diversity of the Mekong River in Laos, for instance, description of *Maesaipsyche mekongensis* found in Luang Prabang Province (Mey 2001). Laudee and Malicky (2017) and Malicky and Laudee (2017) described *Pseudoleptonema tansoongnerni* Malicky & Laudee, 2017, *Pseudoneureclipsis arael* Malicky & Laudee, 2017, *P. hamabiel* Malicky & Laudee, 2017, and *Setodes marianu* Malicky & Laudee, 2017 as new and listed Trichoptera from the Li Phi falls along Mekong River in the southern Laos. Recently, Malicky and Laudee (2019) described 15 new species of caddisflies from tributaries of the Mekong River in Laos.

In this study, we focus on three caddisfly genera in Laos – *Ecnomus* McLachlan, *Polycentropus* Curtis, and *Psychomyia* Latreille. There are eight species of *Ecnomus* reported from Laos, including *E. alkaios* Malicky & Chantaramongkol, 1997, *E. alkmene* Malicky & Chantaramongkol, 1997, *E. androgeos* Malicky, 1997, *E. amphitryon* Malicky, 1997, *E. volovicus* Malicky & Chantaramongkol, 1993, *E. caesar* Malicky & Chaibu in Malicky et al. 2000, *E. dikla* Malicky, 2009, and *E. thamar* Malicky & Laudee in Malicky, 2009 (Malicky 2010; Laudee and Malicky 2017). So far, only two species of *Polyplectropus*, *P. menna* Malicky & Chantaramongkol, 1993 and *P. ammonios* Malicky, 2009, have been recorded in Laos. In *Psychomyia*, seven species have been found from Laos, including *Ps. thienemanni* Ulmer, 1951, *Ps. chompu* Malicky & Chantaramongkol, 1993, *Ps. arthit* Malicky & Chantaramongkol, 1993, *Ps. anteia* Malicky, 1997, *Ps. andromache* Malicky, 1997, *Ps. andromeda* Malicky, 1997, and *Ps. muriel* Malicky & Laudee, 2019 (Malicky 2010; Malicky and Laudee 2019).

Considering the overall diversity of the Mekong River and its tributaries and the under-investigated caddisfly fauna of this region, many new species records and descriptions are expected. This article adds four new species from the Mekong River and its tributaries to the list of Trichoptera in Laos.

## Materials and methods

Adult caddisfly specimens were collected with a UV pan light trap (12 V, 10 W) operated along streams and the river overnight at the locations indicated below. Collected specimens were preserved in 70% ethanol, and caddisflies were later manually sorted from other insects. For species-level identifications, the male genitalia were observed under a stereomicroscope. For this purpose, the male genitalia from a specimen from each new species were dissected out. Muscle tissue was macerated by heating in 10% KOH at 60 °C for 30–60 minutes and then soaking in a detergent solution. Drawings were initially made in pencil using a compound microscope equipped with a drawing tube and used to produce the final vector graphics in Adobe Illustrator software.

Holotypes and paratypes are stored in 70% ethanol and deposited in Princess Maha Chakri Sirindhorn Natural History Museum, Prince of Songkla University, Hat Yai Campus, Hat Yai District, Songkhla Province, Thailand (PSUNHM). Some paratypes are deposited in the collection of Hans Malicky (CHM), the Clemson University Arthropod Collection (CUAC), and the National Museum, Prague, Czech Republic (NMPC). Terminology for genitalic structures for different genera follows that of Cartwright (1994) for the genus *Ecnomus*, Schmid (1997) for the genus *Psychomyia* and Chamorro and Holzenthal (2011) for the genus *Polyplectropus*.

## Systematics

### Ecnomus petchanaae

Taxon classificationAnimaliaTrichopteraEcnomidae

Laudee & Malicky sp. nov.

1292136E-4EBC-5481-968F-185FD740F3F7

http://zoobank.org/61596C7B-1396-41B1-9DC1-60E344674F81

Figure 1

#### Diagnosis.

The male genitalia of *E. petchanaae* sp. nov. are similar to *Ecnomus gapit* Cartwright, 1994, *E. yuleae* Cartwright, 1994, *E. dares* Malicky, 2000, and *E. perseis* Malicky, 2008 described from Borneo. The superior appendages of all these species, including the new species, are particularly large and the subapical part of the superior appendages is covered by numerous spiny setae. However, *E. petchanaae* sp. nov. can be distinguished by the shape of its superior appendages, which, in lateral view, are slender and club-shaped, but basally broad in *E. gapit*, *E. yuleae*, *E. dares*, and *E. perseis*. In addition, in ventral view of the outer surface of the inferior appendages of the new species is crescent-shaped, whereas they are curved and claw-shaped in *E. gapit*, *E. yuleae*, *E. dares*, and *E. perseis*.

**Figure 1.**
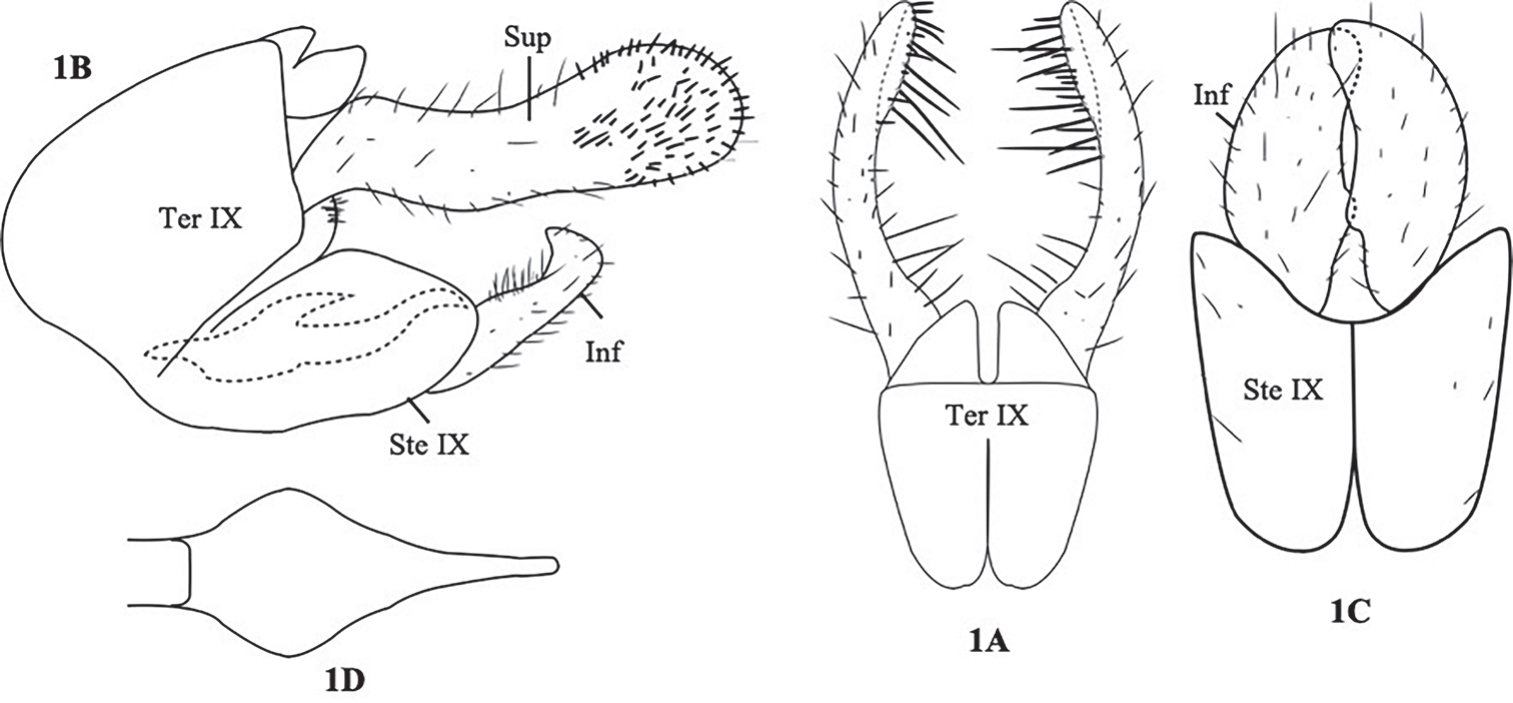
*Ecnomus petchanaae*, sp. nov. Male genitalia. **A** Segment IX and superior appendages, dorsal **B** segments IX and superior appendages, left lateral **C** segment IX and inferior appendages, ventral **D** phallus tip, ventral. Ter IX = tergum IX, Ste IX = sternum IX, Sup = superior appendage, Inf = inferior appendage.

#### Description.

Adult, male, length of each male forewing 5.6–6.0 mm; color in alcohol of head, thorax, forewings, abdomen, and legs brown. Male genitalia as in Figure 1A–D. Tergum IX somewhat square, anterior margin truncated, posterior margin bilobed in dorsal view (Fig. 1A); trapezoid and rounded anterodorsally in lateral view (Fig. 1B). Sternum IX ovoid in lateral view (Fig. 1B); rectangular with ¼ concave incision posteriorly, bilobed and rounded anteriorly in ventral view (Fig. 1C). Superior appendages long, slender, with expanded base, curved inward posteriorly with numerous long spiny setae subapically in dorsal view (Fig. 1A); in lateral view, superior appendages, relatively large, long, slightly curved upward, bulb-like apically, with numerous spiny setae (Fig. 1B). Inferior appendages tubular, bent inward, beak-like apically in lateral view (Fig. 1B); in ventral view, crescent-shaped, with a submediate knot, overlapping each other subapically (Fig. 1C). Phallus long, tubular, curved upward, pointed apex with dorsal process in lateral view (Fig. 1B); in ventral view, bulb-like, with pointed apex (Fig. 1D).

#### Type material.

***Holotype.* Male. Laos: Pakse Province**: Paksong, Vang Ngao River, 15°11'37"N, 106°06'40"E, elev. 920 m, 7.iv.2019, Pongsak Laudee. (PSUNHM). ***Paratypes***: same data as the holotype, 3 males: 1 male (PSUNHM), 1 male (CHM), 1 male (NMPC).

#### Etymology.

The species epithet honors Mrs Kanchanaluk Petchana, Director of Administration and Strategic Development Division, Prince of Songkla University, Surat Thani Campus.

### Ecnomus boonsawaengae

Taxon classificationAnimaliaTrichopteraEcnomidae

Malicky & Vongsombath sp. nov.

BCFCC471-AED1-59DA-AE84-C09B8E4BF563

http://zoobank.org/A970D2D6-D505-4A72-B693-D2C6DC131325

Figure 2

#### Diagnosis.

The male genitalia of *E. boonsawaengae* sp. nov. are similar to *E. aktaion* Malicky & Chantaramongkol, 1997 and *E. uttu* Malicky & Chantaramongkol, 1993. In these species, the superior appendages are particularly long and slender with a basoventral process on the superior appendage. However, *E. boonsawaengae* sp. nov. can be easily distinguished by the shape of the inferior appendages. In lateral view, the inferior appendages are tubular with a subapical concave incision and truncated apex in *E. boonsawaengae* sp. nov., but in *E. aktaion* and *E. uttu* the inferior appendages are somewhat triangular and trapezoidal, respectively, and with a pointed apex. In addition, each inferior appendage in *E. boonsawaengae* sp. nov. has a process, in ventral view, which is lacking in *E. aktaion* and *E. uttu*.

#### Description.

**Figure 2.**
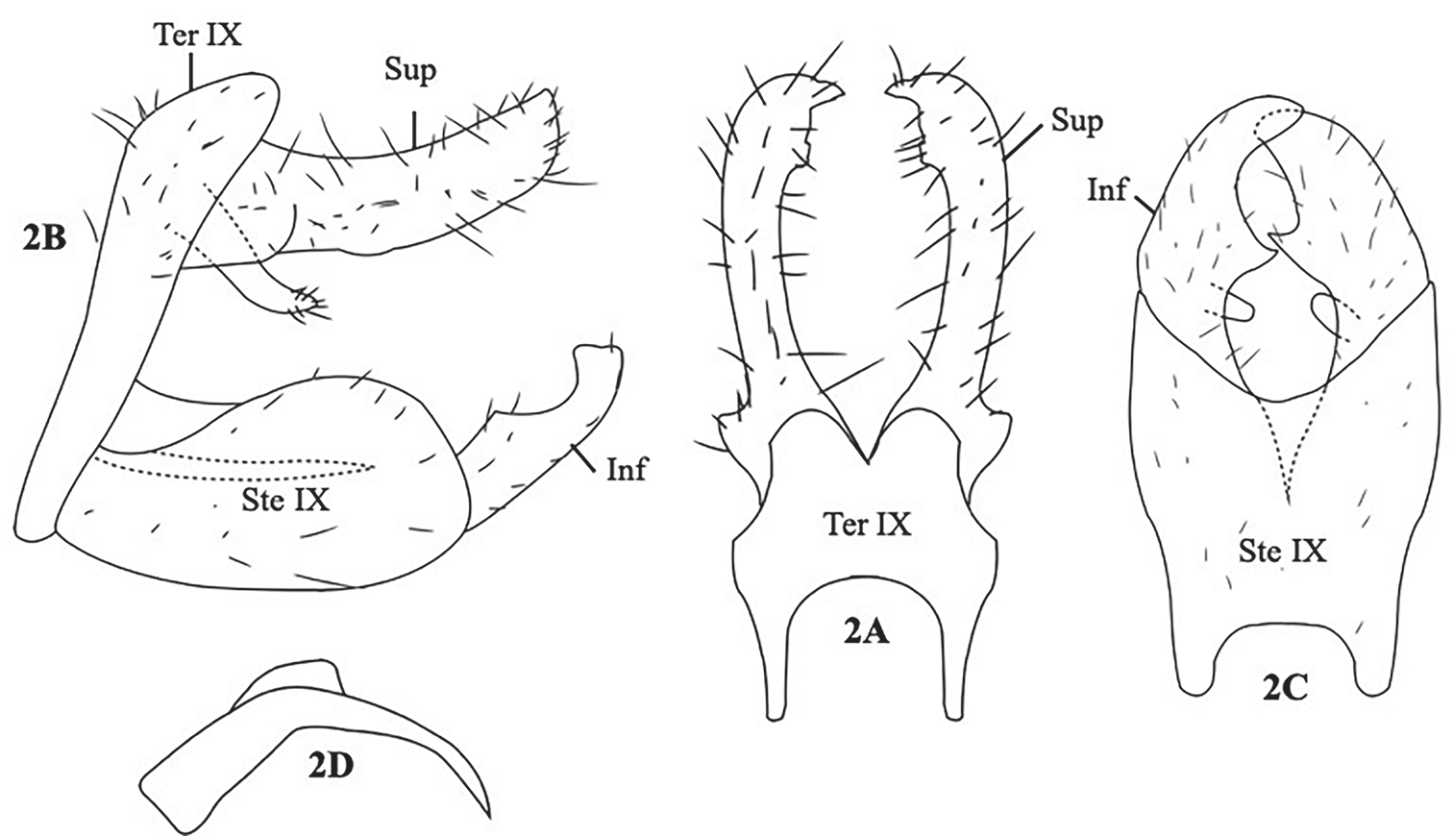
*Ecnomus boonsawaengae*, sp. nov. Male genitalia. **A** Segment IX and superior appendages, dorsal **B** segment IX and superior appendages, left lateral **C** segment IX and inferior appendages, ventral **D** phallus, lateral. Ter IX = tergum IX, Ste IX = sternum IX, Sup = superior appendage, Inf=inferior appendage.

Adult, male, length of each male forewing 4.0 mm; color in alcohol of head, thorax, forewings, abdomen, and legs grayish brown. Male genitalia as in Figure 2A–D. Tergum IX in dorsal view bilobed posteriorly, U-shaped ½ incision anteriorly (Fig. 2A); in lateral view, tergum IX narrow, expanded dorsally (Fig. 2B). Superior appendages tubular, base with lateral lobe, slightly bent apically to form beak-like apex in dorsal view (Fig. 2A); in lateral view, tubular, truncated apically (Fig. 2B). Basoventral projection of superior appendage tubular with setae apically in lateral view. Sternum IX in lateral view chicken-drumstick-like and rounded apically (Fig. 2B); in ventral view, trapezoid, slightly expanded apically, with shallow U-shaped incision anteriorly and shallow V-shaped incision posteriorly (Fig. 2C). In lateral view, the inferior appendages tubular, with concave incision subapically, truncated apex (Fig. 2B); in ventral view, claw-like, with process basodorsally (Fig. 2C). Phallus sickle-like, with lobe mesodosally in lateral view (Fig. 2D).

#### Type material.

***Holotype.* Male. Laos: Pakse Province**: Paksong, E-Tu Waterfall, 15°11'25"N, 106°06'14"E, elev. 900 m, 7.iv.2019, Pongsak Laudee. (PSUNHM). ***Paratypes***: same data as holotype, 2 males: 1 male (PSUNHM), 1 male (CHM).

#### Etymology.

The species epithet honors Mrs Wasana Boonsawaeng, Vice Dean of Faculty of Science and Industrial Technology, Prince of Songkla University, Surat Thani Campus.

### Polyplectropus proukaewi

Taxon classificationAnimaliaTrichopteraPolycentropodidae

Malicky & Seetapan sp. nov.

FE3E7270-2DB5-54A6-B555-95DABF316A4C

http://zoobank.org/180D25B8-A9AC-4B3D-BE3C-C2D884F460EF

Figure 3

#### Diagnosis.

The male genitalia of *P. proukaewi* sp. nov. are similar to those of *P. daimong* Oláh & Johanson, 2010 from Vietnam. In both species, the dorsal branch of the inferior appendages forms a hooked-like process. However, *P. proukaewi* sp. nov. can be distinguished by the considerably shorter distance of the distal processes of the dorsal branch of the inferior appendages to their bases compared to *P. daimong*, in which this distance is considerably longer. Additionally, in the new species, the length of the distal processes in lateral view equals half of the length of inferior appendages, whereas the length of the distal processes equals the length of inferior appendages in *P. daimong*.

#### Description.

**Figure 3.**
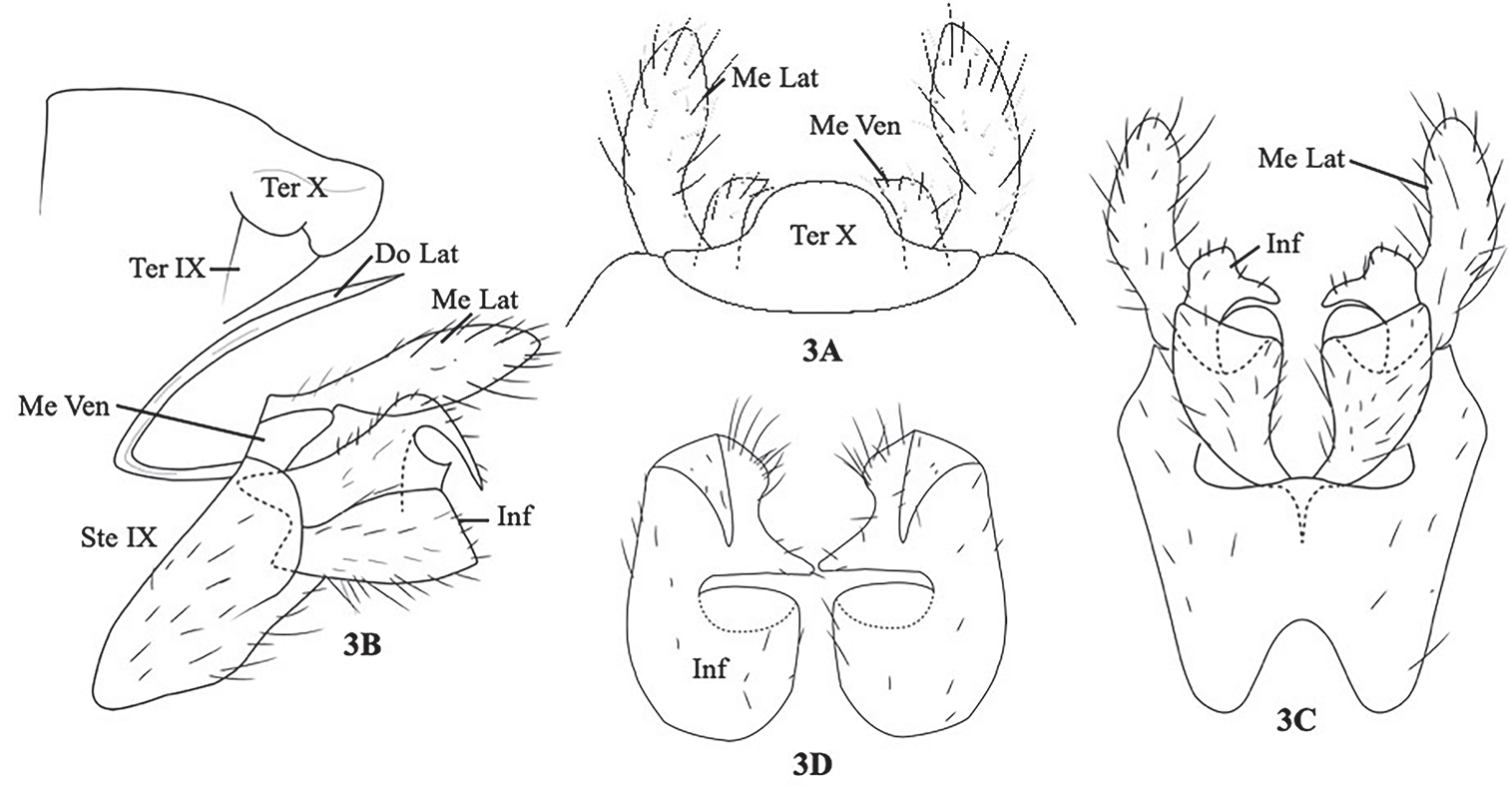
*Polyplectropus proukaewi*, sp. nov. Male genitalia. **A** Segment X, dorsal **B** segments IX–X, left lateral **C** inferior appendages, ventral **D** inferior appendages, caudal. Ter IX = tergum IX, Ste IX = sternum IX, Ter X = Tergum X, Do Lat = dorsolateral processes of preanal appendages, Me Lat = mesolateral processes of preanal appendages, Me Ven = mesoventral processes of preanal appendages.

Adult, male, length of each male forewing 6.5–7 mm; color in alcohol of head, thorax, forewings, abdomen, and legs brown. Male genitalia as in Figure 3. Tergum IX triangular, underneath Tergum X in lateral view (Fig. 3B). Sternum IX subtriangular and rounded anteriorly in lateral view (Fig. 3B); in ventral view, hexagonal, with V-shaped incision anteriorly (Fig. 3C). Tergum X hat-shaped in dorsal view (Fig. 3A), B-shaped in lateral view (Fig. 3B). Dorsolateral process of preanal appendages sclerotized, needle-like, straight and horizontal subbasally then recurving upward subapically, distal end pointed in lateral view (Fig. 3B). Mesolateral process of preanal appendages thumb-like, with setae in dorsal view (Fig. 3A); in lateral view leaf-like, with setae (Fig. 3B). Mesoventral processes of preanal appendages short, finger-like, with setae in dorsal view (Fig. 3A); in lateral view, subtriangular, with setae, underneath base of mesolateral processes (Fig. 3B). In lateral view, inferior appendages trapezoidal, with V-shaped incision anteriorly, each dorsal branch of inferior appendages with hook-like processes posterodorsally, half the length of inferior appendages (Fig. 3B). In ventral view, each ventral branch of inferior appendages subtriangular, each dorsal branch of inferior appendages with “bird head-like” dorsal branch of inferior appendages posteriorly (Fig. 3C). In caudal view, inferior appendages oval, with triangular processes dorsally and nose-like process mesally (Fig. 3D)

#### Type material.

***Holotype.* Male. Laos: Pakse Province**: Paksong, Vang Ngao River, 15°11'37"N, 106°06'40"E, elev. 920 m, 7.iv.2019, Pongsak Laudee. (PSUNHM). ***Paratypes***: same data as holotype, 16 males: 6 males (PSUNHM), 5 males (CHM), 5 males (NMPC).

#### Etymology.

The species epithet honors Dr Nitat Proukaew, Assistant Professor in the Faculty of Science and Industrial Technology, Prince of Songkla University, Surat Thani Campus.

### Psychomyia srichanai

Taxon classificationAnimaliaTrichopteraPsychomyiidae

Laudee & Malicky sp. nov.

A6C8690C-1FDC-5A10-A837-EB47D8105F34

http://zoobank.org/F525EC47-7B79-4FFF-911A-97FD1EC90519

Figure 4

#### Diagnosis.

The male genitalia of *Ps. srichanai* sp. nov. are similar to those of three other *Psychomyia* species described from Thailand, *Ps. amor* Malicky & Chantaramongkol, 1997, *Ps. amphiaraos* Malicky & Chantaramongkol, 1997 and *Ps. monto* Malicky & Chantaramongkol, 1993, as well as of *Ps. sonlana* Oláh & Malicky, 2010 from Vietnam. Differences are mainly seen in the structure of the dorsal branches of the harpagones and apical sclerite. The dorsal branch of each harpago in *Ps. srichanai* sp. nov. is divided into two branches. The outer branch curves downward and bifurcates apically, whereas the inner branch is long, curved upward, and apically pointed. In *Ps. amphiaraos* and *Ps. monto*, the outer dorsal branch does not bifurcate. The outer dorsal branches of the harpagones are also bifurcated in *Ps. amor* and *Ps. sonlana*. However, only in *Ps. srichanai* sp. nov. are they are bent outward at 90°. The apical sclerite is apically pointed and discernable in *Ps. amphiaraos*, *Ps. monto*, *Ps. amor*, and *Ps. sonlana*, but indiscernible in *Ps. srichanai* sp. nov.

#### Description.

**Figure 4.**
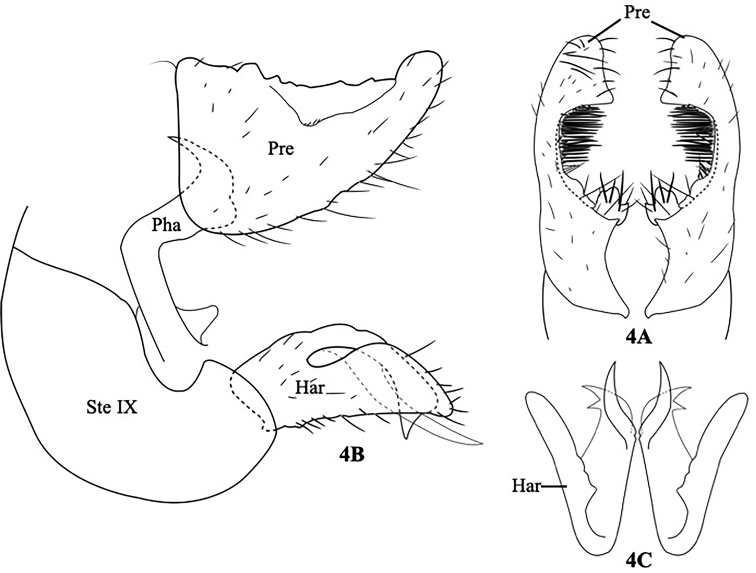
*Psychomyia srichanai*, sp. nov. Male genitalia. **A** Segment IX, preanal appendages and harpago, lateral **B** preanal appendages, dorsal **C** harpago, ventral. Har = harpago, Pre = preanal appendage, SIX = sternum of segment IX, Pha = phallus.

Length of each male forewing 3.0–4.0 mm; color in alcohol of head, thorax, forewings, abdomen, and legs yellow brown. Male genitalia as in Figure 4. Preanal appendages crescent-shaped, with U-shaped incision inward mediately, with long setae inward medially, rounded apically in dorsal view (Fig. 4A); in lateral view, preanal appendages large, triangular, rounded apically (Fig. 4B). Sternum IX crookneck-squash-shaped, pointed dorsally, truncated apically in lateral view (Fig. 4B). Harpagones crab-claw-shaped; dorsal branch of each harpago divided into two branches, the outer branch curved downward and bifurcated apically, the inner branch long, curved upward, and pointed apically; ventral branch of each harpago triangular, curved dorsally, rounded apically in lateral view (Fig. 4B). In ventral view, outer dorsal branches of harpago tubular, bent outward subapically, bifurcated apically; ventral branches of harpago tubular, rounded apically (Fig. 4C). Phallus long, tubular, bent 90° medially, curved upward subapically, distal apex in lateral view (Fig. 4B).

#### Type material.

***Holotype.* Male. Laos: Luang Prabang Province**: Elephant Camp, Mekong River, 20°01'46"N, 102°13'13"E, elev. 280 m, 2.iii.2019, Pongsak Laudee. (PSUNHM). ***Paratypes***: same data as holotype, 40 males: 10 males (PSUNHM), 10 males (CHM), 10 males (NMPC), 10 males (CUAC).

#### Etymology.

The species epithet honors Prof. Dr Teerapol Srichana, Director of the Research and Development Office, Prince of Songkla University, Hat Yai Campus.

## Discussion

**Figure 5.**
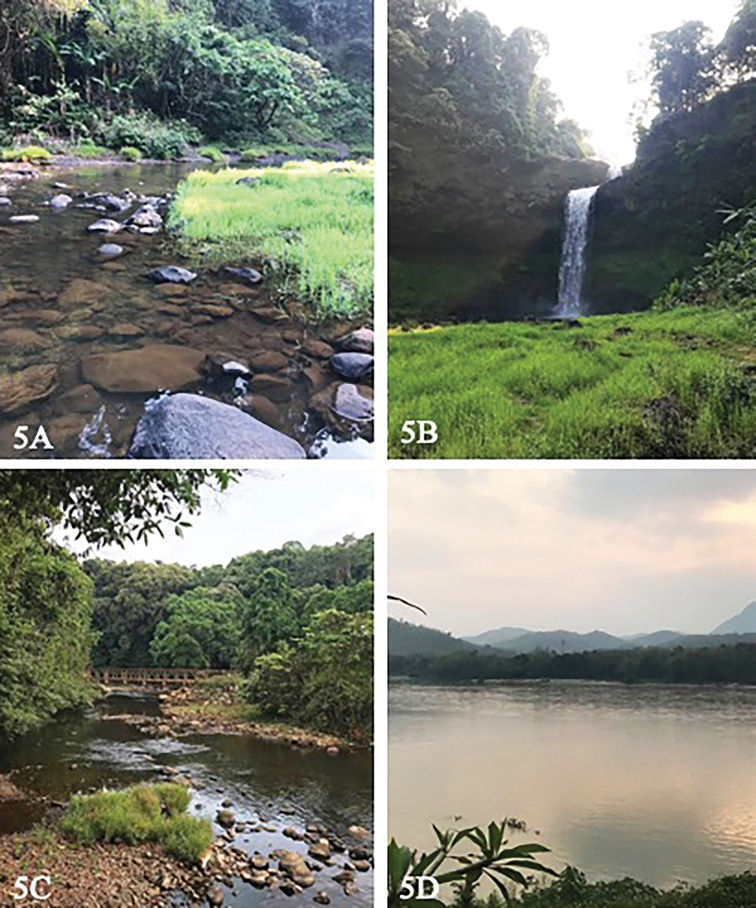
Study sites from Mekong river and its tributaries. **A, B** E-Tu Waterfall, Paksong, Pakse Province **C** Vang Ngao River, Paksong, Pakse Province **D** the Mekong River, Luang Prabang Province.

*Ecnomus petchanaae* sp. nov., *E. boonsawaengae* sp. nov. and *P. proukaewi* sp. nov. were collected from a stream and waterfall on Bolaven Plateau, southern Laos. Here, forest type is montane evergreen rainforest. The three new species are rhithral species, which live in small streams where the substrate is dominated by boulders and cobblestones (Fig. 5A–C). Along with the eight previously recorded species of *Ecnomus*, there are now 10 species belonging to this genus are now known in Laos; of these, four species, including the new species, are apparently endemic to the country (Malicky 2010; Laudee and Malicky 2017). *Ecnomus* species not only occur in rhithron stream zones but also are found in potamon stream zones (Laudee and Malicky 2017). Three species of *Polyplectropus*, including the new species, are now recorded from Laos. Among these, two species are reported only from Laos (Malicky 2010). *Psychomyia srichanai* sp. nov. was collected from main river channel of the Mekong River in Luang Prabang Province, northern Laos (Fig. 5D). This is a potamon species that lives in main Mekong River. *Psychomyia srichanai* sp. nov. is in *P. capillata* species group according to the charecters of the group as diagnosed by Malicky and Chantaramongkol (1993). In total, eight species of *Psychomyia* have been reported from Laos, of which four species, including the new species, are reported only from Laos (Malicky 2010).

## Supplementary Material

XML Treatment for Ecnomus petchanaae

XML Treatment for Ecnomus boonsawaengae

XML Treatment for Polyplectropus proukaewi

XML Treatment for Psychomyia srichanai
